# Protein bomarkers Ki67, HOXC10 and HOXC11 for the prediction of response to endocrine treatment in breast cancer

**DOI:** 10.1186/1753-6561-9-S1-A52

**Published:** 2015-01-14

**Authors:** YH Soon, F Bane, E Hughes, LS Young

**Affiliations:** 1 Royal College of Surgeons in Ireland, 123 St. Stephen’s Green, Dublin 2, Ireland; 2 Endocrine Oncology Research, Department of Surgery, Royal College of Surgeons in Ireland, 123 St. Stephen’s Green, Dublin 2, Ireland

## Background

Breast cancer is a common malignant tumour prevalent in Ireland and rest of the world. Although breast cancer screening and treatment has dramatically improved patient prognosis, it is still the leading cause of cancer-related death for women. One of the major bases for breast cancer treatment is patient’s receptor status: ER, PR, HER2 positive or negative. Immunohistochemistry (IHC) is a technique that diagnoses abnormal cells, which uses antibodies to detect antigens (proteins) of interest in a biological tissue. IHC is widely used in hospitals and laboratories, for screening patient receptor status. In this study, IHC was performed on breast tumour samples obtained at Beaumont Hospital to assess 3 protein biomarkers Ki67, HOXC10 and HOXC11. Aim: This study aimed to use IHC technique to: 1. Optimize HOXC11 staining for use on TMA (Tissue MicroArray) 2. Assess Ki67 staining in a pre-stained TMA 3. Stain full face sections from primary and matched metastatic tissue for HOXC10.

## Methods

To perform Immunohistochemistry, 3 stages were carried out: TMA preparation, Antigen Retrieval and DAKO staining. The stained tissue samples were scored using Allred Scoring System (for HOXC10 and HOXC11) and Aperio Imagescope (for Ki67) to determine positivity and intensity of its stain. Each patient was then categorised into stain-positive and stain-negative groups, as the binary input data for analysis. Stata 10 software program was used to compare protein profile of the patients with already-existing patient database which also contains information on patient prognosis. Wilcoxon statistical analysis was performed to compare patient outcome based on patient receptor status and protein profile.

## Results

1. HOXC11 staining was successful, but inconsistent. 2. HOXC10 is expressed in primary breast tumour; however this expression is lost in matched metastatic tissue. 3. Ki67 positive patients have faster tumour recurrence (p=0.0051). 4. Luminal breast cancer type (ER+ve, PR +ve/-ve and HER2-ve) with positive Ki67 status have faster tumour recurrence (p=0.0004).

## Conclusions

1.HOXC11 staining optimization is required for a robust staining protocol. Investigating efficacy of other antigen retrieval methods is required such as using a pressure cooker. 2. HOXC10 expression was reduced in metastatic breast cancer, which corroborates findings in the Oesterrich lab [1] 3. Ki67 positive status was shown to be associated with faster tumour recurrence and its sub-categorization of Luminal type is agreement with Hughes et al [2] IHC optimization is important for robust understanding of new proteins in cancer, which may help improve personalized medicine and enhance optimal treatment even further.
